# The acceptability, feasibility and impact of a lay health counsellor delivered health promoting schools programme in India: a case study evaluation

**DOI:** 10.1186/1472-6963-12-127

**Published:** 2012-05-25

**Authors:** Divya Rajaraman, Sandra Travasso, Achira Chatterjee, Bhargav Bhat, Gracy Andrew, Suraj Parab, Vikram Patel

**Affiliations:** 1 Division of Epidemiology, St John’s Research Institute, Bangalore, 560034, India; 2 Sangath, Alto Porvorim, Goa, India; 3 Centre for Global Mental Health, London School of Hygiene & Tropical Medicine, London WC1E 7HT, United Kingdom

## Abstract

****Background**:**

Studies in resource-limited settings have shown that there are constraints to the use of teachers, peers or health professionals to deliver school health promotion interventions. School health programmes delivered by trained lay health counsellors could offer a cost-effective alternative. This paper presents a case study of a multi-component school health promotion intervention in India that was delivered by lay school health counsellors, who possessed neither formal educational nor health provider qualifications.

****Methods**:**

The intervention was based on the WHO’s Health Promoting Schools framework, and included health screening camps; an anonymous letter box for student questions and complaints; classroom-based life skills training; and, individual psycho-social and academic counselling for students. The intervention was delivered by a lay school health counsellor who had attained a minimum of a high school education. The counsellor was trained over four weeks and received structured supervision from health professionals working for the implementing NGO. The evaluation design was a mixed methods case study. Quantitative process indicators were collected to assess the extent to which the programme was delivered as planned (feasibility), the uptake of services (acceptability), and the number of students who received corrective health treatment (evidence of impact). Semi-structured interviews were conducted over two years with 108 stakeholders, and were analysed to identify barriers and facilitators for the programme (feasibility), evaluate acceptability, and gather evidence of positive or negative effects of the programme.

****Results**:**

Feasibility was established by the high reported coverage of all the targeted activities by the school health counsellor. Acceptability was indicated by a growing number of submissions to the students’ anonymous letter-box; more students self-referring for counselling services over time; and, the perceived need for the programme, as expressed by principals, parents and students. A minority of teachers complained that there was inadequate information sharing about the programme and mentioned reservations about the capacities of the lay health counsellor. Preliminary evidence of the positive effects of the programme included the correction of vision problems detected in health screening camps, and qualitative evidence of changes in health-related knowledge and behaviour of students.

****Conclusion**:**

A task-shifting approach of delegating school health promotion activities to lay school health counsellors rather than education or health professionals shows promise of effectiveness as a scalable model for promoting the health and well being of school based adolescents in resource constrained settings.

## **Background**

Approximately a third of the Indian population is between the ages of 10–24 years [1]. Young people in India face a range of sexual and reproductive health risks such as HIV infection, early marriage and sexual abuse [2]. They are also affected by the double burden of under and over-nutrition [3,4], while coping with social, academic and mental health concerns [5]. Since the World Health Organization (WHO) Ottawa Charter of 1986, there has been increasing recognition of the need to promote health in the settings in which people live, learn, work and love [6-8]. Given the amount of time young people spend in formal education, schools are an ideal setting for promoting adolescent health [9,10]. The government of India, in its National Health Policy, has identified schools as a key location for improving the health of young people, through provision of information and health services [11]. The WHO Health Promoting Schools (HPS) model was endorsed by a Government of India committee in 2007 [12]. Some of the distinguishing features of a HPS are that it involves all significant concerned stakeholders; strives to provide a safe and healthy environment; promotes skills based health education; promotes access to health services; promotes adoption of health promoting policies & practices; and, ultimately strives to improve the health of the community [9]. Health promoting schools go beyond the conventional model of simply providing information about health; they aim to create a setting where the school environment, policies, institutional culture and linkages with external partners all contribute to improving the health and education outcomes of students. This approach is thought to be particularly conducive for empowerment and health literacy within a society [13].

Across India, government and NGOs are working in partnership to develop, implement and evaluate different models for school health promotion [14-19]. However, the evidence for their acceptability, feasibility and effectiveness is limited [2]. An ongoing debate for the design of school health programmes is the choice of human resource delivery model. A range of studies in different parts of the world have attempted to assess whether teacher or peer led models of health education are more effective. To date the evidence remains inconclusive [20]. The sustainability of either of these models may be questionable in resource constrained settings, given competing pressures of time for both teachers and peers, and a severe global shortage of qualified school teachers [21].

In the context of the global shortage of human resources for health, the World Health Organization has endorsed the concept of ‘task-shifting’, which involves the delegation of tasks and responsibilities from more specialised to less specialised cadres; successful implementation of task-shifting requires clearly defined roles, as well as robust systems of supervision and referral [22]. In settings where adolescent school health promotion is constrained by a shortage of teachers, health educators and qualified counsellors to deliver programmes, a lay school health counsellor could provide a scalable option for implementing health promotion in schools. This paper reports on a case study of a lay school health counsellor led health promotion programme in secondary schools in Goa, India. The aim of the case study was to evaluate the programme’s potential for scale up, in terms of it feasibility, acceptability, and preliminary evidence of impact.

## **Methods**

### **Study setting**

The study was carried out in Goa, a state on the west coast of India. Goa has a population of about 1.45 million, and a higher level of urbanization and school enrolment than the Indian national average. The programme was set in ten government aided secondary schools in rural and peri-urban areas which are likely to be representative of schools serving low income populations in urban and semi-urban areas of India.

### **The intervention**

The **S**chool **H**e**A**lth **P**romotion and **E**mpowerment (SHAPE) programme was developed by a Goa-based NGO, Sangath (http://www.sangath.com), building on previous experience in adolescent mental and sexual health interventions that were led by teachers and peers [23], and a pilot school health promotion project delivered by lay School Health Counsellors (SHCs) in four schools in Goa in 2008–9. The intervention, which was targeted to male and female students from 5^th^ to 12^th^ grades in the age range of 9–17 years old, was aligned with the HPS approach. The activities included annual visual assessment and nutritional screening camps for all students; an anonymous letter box for students to voice their questions and concerns (Speak Out Box); classroom-based life skills training and individual counselling for students. A description of the intervention components is provided in Table 1.

**Table 1:** The Health Promoting Schools Intervention delivered by Lay School Health Counsellors

**UNIVERSAL (SCHOOL-WIDE) LEVEL**	
*School Mapping & Needs Assessment*	Mapping to assess the infrastructure, health environment and health resources available. A structured questionnaire was administered to school management, teachers, students and parents to identify health and wellbeing priorities. This information was used to tailor the intervention for each school’s needs.
*Speak-Out Box*	A letter box mounted on a wall in an easily accessible area in which school members could make anonymous submissions on any health, social or other school related concern. The SHC reviewed submissions on a weekly basis and followed these up as appropriate.
*Health Camps *	Visual Screening and BMI Assessments. SHCs were trained to take weight and height measurements and to conduct visual screening to identify possible refractory errors and colour blindness. Body Mass Index was estimated, and students identified with possible nutrition or visual problems were given appropriate advice/referral.
**CLASS LEVEL**	
*Life Skills Education*	The Life Skills classroom programme was developed using international and national resources. The programme was delivered over one period (35–40 minutes) per class per week. The sessions were designed to be interactive and activity based. They covered physiological and sexual and reproductive health; psycho-social issues/mental health; and, effective learning techniques.
**INDIVIDUAL LEVEL**	
*Individual Counselling*	Face to face counselling for students who were self-referred or referred by a teacher or principal. In the first year, a clinical psychologist conducted the counselling sessions in the presence of the SHC. In the second year, the SHC provided counselling to the students, with ongoing supervision and support from the NGO staff.

*In addition to the activities detailed in the table, the SHC coordinated a number of workshops for the different stakeholder groups. These included nutrition, parenting, teaching methods, and development of healthy school policies. The workshops were delivered by the NGO and partners.

The intervention was implemented by female SHCs between 2009 and 2011. Every school had one full-time SHC, who was nominated by the participating school, but paid by the NGO. Some of the SHCs were alumni of the schools in which they were placed. Before the start of the intervention, the SHCs received 40 days of training from the implementing NGO to acquire the knowledge and skills considered necessary to fulfil their roles. Their responsibilities included: coordinating with stakeholders and convening meetings; conducting nutritional and visual assessments of all students; reviewing and acting upon Speak Out Box submissions; delivering the classroom based life skills programme; providing individual counselling to students; and, record-keeping. In the first year of the intervention, the SHCs received supervision from a clinical supervisor (trained clinical psychologist) in weekly group meetings at the NGO office and bi-monthly on-site school visits. In the second year of the programme, the supervision was scaled down to bi-monthly group meetings at the NGO office and monthly school supervisory visits. The individual counselling was conducted jointly by a clinical supervisor and the SHC in the first year of the intervention, as part of on the job training. In the second year, the SHCs were encouraged to counsel students on their own, only referring difficult cases to the clinical supervisor.

Each school had a School Health Promotion Advisory Board (SHPAB), which included the school principal, the SHC, one teacher, two parents, and a male and female student. The SHPAB was scheduled to meet once in three months, and its purpose was to tailor the programme for the school’s needs, provide input on all activities, monitor progress, and relay information about the programme and targets to the stakeholder groups.

The intervention also included facilitating the development and implementation of healthy school policies (on bullying and violence, substance use, and inclusive education). This activity involved both the SHC and programme staff from the NGO, and it is not described in this paper.

### **Study design**

A mixed methods case study approach was used for this evaluation, with the unit of analysis being the pilot school health promotion programme. The case study design is a methodology that allows for a phenomenon to be described and interpreted in its context, using multiple of data sources [24]. It is a particularly versatile approach, allowing for variety in the selection of the unit of analysis and a range of methods [25]. The case study is considered useful in health sciences research, as it provides flexibility and rigour for developing and testing theory, evaluating programmes, and developing interventions [26]. The aims of this study were to assess the feasibility and acceptability of the intervention and gather preliminary evidence of impact [Table 2. The study aimed to generate evidence for whether the pilot intervention showed potential for further evaluation of effectiveness and the descriptive approach of the study is consistent with the formative and piloting research phases of the UK MRC framework for complex interventions [27]. Nine schools were evaluated over a two year period; in one school, the intervention started only in the second year.

**Table 2:** Evaluation framework for the SHAPE intervention

**Theme**	**Research Questions**	**Quantitative Process Indicators**	**Qualitative themes**
*Feasibility*	Was it possible to deliver the programme as planned?	Proportion of target activities (SHPAB meetings, health camps, and life skills sessions) delivered	Programme coordinator, SHC, school management, teacher and student perceptions of barriers and facilitators
	What were the major barriers and facilitators?		
*Acceptability*	Were the services thought to be adding value?	number of submissions to the Speak Out Box	School management, teacher, student, and parent perceptions of need for and appropriateness of programme, and of adequacy of SHC skills
	Were the services used?	number of students accessing individual counselling	
	Was the SHC considered capable of delivering the services?		
*Impact*	In what ways did the programme improve the health and wellbeing of the students and influence the overall environment of the school?	Number of students who attended health camps, were identified as having problems, and sought follow up care	Qualitative evidence of impact from interviews with all stakeholders

### **Quantitative programme monitoring data**

Programme monitoring data were collected over two years to assess whether the programme was delivered as planned (indicating feasibility), to measure the uptake of services (indicating acceptability), and to determine the number of students who received treatment following health screening camps (evidence of impact). The quantitative process indicators included the number of activities delivered, the number of participants reached by different activities, and the number of health or other problems identified and rectified through the various activities. These data were recorded by the SHCs in their daily monitoring registers, and were reviewed for accuracy and completeness on a weekly basis by a research officer. The data were subsequently entered into an electronic database, and cleaned and analysed in SPSS [28]. The coverage of the programme was estimated as the proportion of the planned activities that was delivered, as per the intervention targets set at the beginning of the year by the programme development team.

### **Qualitative semi-structured interviews**

The purpose of the semi-structured interviews was to capture the range of experiences and opinions relating to the programme in its first and second year, in order to identify barriers and facilitators (feasibility); assess perceived utility of the various programme activities (acceptability); and, gather evidence of positive or negative effects of programme activities (impact). Using a quota sampling strategy, a total of 108 semi-structured interviews were conducted with SHCs, programme coordinators and clinical supervisors from the implementing NGO, school principals, teachers, and students. All programme staff and school principals were invited to participate. As a large number of teachers and students were reached by the programme, only a subset in these groups were interviewed; in each school, one teacher and a male and female student were selected from the SHPAB (school advisory board), and a second teacher was randomly selected from the teachers’ list for inclusion into the study. Students were randomly selected, after stratifying for school, gender, and utilization of the counselling service. Random sampling was used in order to avoid the bias of best or worst cases being nominated for interview by principals or SHCs. The large number of interviews helped to ensure that the range of experiences and opinions was captured across schools and stakeholder groups. The details of the semi-structured interview sample are shown in Table 3.

**Table 3:** Semi-structured interview sample

**Stakeholder Group (Sample Frame)**	**2009-10**	**2010-11**	**Total**
Male student (member of SHPAB)	9	4	13
Male student (from those who attended counselling sessions)	9	4	13
Female student (member of SHPAB)	9	4	13
Female student (from those who attended counselling sessions)	9	4	13
Teacher (Teaching staff list)	9	-	9
Teacher (member of SHPAB)	9	4	13
Parent (member of SHPAB)	*-*	4	4
Principal (all)	9	4	13
SHC (all)	9	4	13
Intervention coordinator (all)	*-*	2	2
Clinical Supervisors (all)	*-*	3	*3*
***TOTAL***	***72***	**37**	***109***

In the first year of the programme, semi-structured interviews were conducted in all the 9 programme schools at the end of the year. In the second year, interviews were conducted in a sub-sample of 4 schools. Semi-structured interviews were conducted by three independent evaluators who were trained and supervised by experienced qualitative researchers. Interviews were conducted in Konkani (the local language) and/or English, depending on the preference of the interviewee. The research protocol received approval from the Sangath institutional ethics review board. Written informed consent was obtained from all participants, and parental consent was taken from parents of the students.

The semi-structured interviews included sections for each of the programme activities, the school health counsellor as an agent of delivery, and the overall programme. Interview guides were tailored for each type of stakeholder, probing for experiences and opinions in each section. Interviews were translated and transcribed for analysis, and Atlas-Ti was used for initial data coding [29]. A framework analysis was conducted: codes were applied for each of the programme activities, the SHC as an agent of delivery and the overall programme, and for the themes of acceptability (‘things liked’, ‘things not liked’), feasibility (‘feasible’, ‘not feasible’, ‘barriers’, ‘facilitators’), impact (‘positive effects’, ‘negative effects’), and suggestions for improvement. An initial set of transcripts was double coded to ensure consistency in applying the codes. The data were synthesised thematically in spread-sheets for each programme activity, and common themes and illustrative examples were identified.

## **Results**

The results are presented in three sub-sections, which report quantitative programme monitoring data and qualitative data from semi-structured interviews to assess: i) feasibility; ii) acceptability; and, iii) evidence of impact.

### **Feasibility**

The SHAPE programme was delivered in 9 schools to 2,105 students in 2009–10, and in 10 schools to 2,199 students in 2010–11. The feasibility of the programme was assessed quantitatively by the proportion of target programme activities that were delivered, and qualitatively in terms of the barriers and facilitators identified by stakeholders. The proportion of target activities delivered in the first year was high, and increased in the second year despite reduced supervision of the SHCs (Table 4). Over the two years, the quarterly SHPAB meetings were held in all schools as scheduled, the visual assessment and health camps were held in all schools as per target, and the completion of the life skills programme increased from 86.6% to 92.2%. The number of students who accessed counselling increased marginally from 122 to 128, but the proportion of students remained steady, between 6.2-6.3% of all students.

**Table 4:** **Coverage of the intervention activities in all schools 2009-11**^**1**^

**School Health Promotion Advisory Board Meetings**						
	**2009**–**2010**			**2010**–**2011**		
	**Target**	**Actual**	**Coverage**	**Target**	**Actual**	**Coverage**
No. of SHPAB meetings	27	27	**100%**	30	30	**100%**
**Health Camps by Sangath**						
	**2009**–**2010**			**2010**–**2011**		
	**Target**	**Actual**	**Coverage**	**Target**	**Actual**	**Coverage**
Visual Assessment	9	9	**100%**	10	10	**100%**
BMI	9	9	**100%**	10	10	**100%**
**Delivery of Classroom Life Skills Education sessions**						
	**2009**–**2010**			**2010**–**2011**		
	**Target**	**Actual**	**Coverage**	**Target**	**Actual**	**Coverage**
Physiological	428	326	**76.2%**	433	394	**91%**
Psychosocial	735	661	**89.9%**	711	651	**91.6%**
Effective learning techniques	350	324	**92.6%**	331	315	**95.2%**
**TOTAL**	1513	1311	**86.6%**	1475	1360	**92.2%**
**School range (Total)**			**(84.6 – 98.9%)**			**(84.4 – 99.3%)**
**Individual Counselling**						
	**2009**–**2010**			**2010**–**2011**		
Number of cases	122			128		
% Students who accessed counselling)	6.2%			6.3%		
School Range (% Students who accessed counselling)	(1.8–13.1%)			(2.3–11.1%)		
Number of follow ups	251			323		
% Sessions led by SHC^2^	18.5%			55%		
School Range (Sessions led by SHC)	(1.8–41.2%)			(30–92.8%)		

^*1*^*Coverage defined as percentage of target achieved. Targets were set a priori by the intervention coordinators*.

^*2*^*Other sessions were led by a clinical psychologist in the presence of the SHC*.

Interviews with SHCs and programme coordinators revealed that in the first year of the intervention, almost half the SHCs did not have a regular class period and/or a classroom allocated for teaching the life skills sessions. Consequently, they had to deliver the classes either after school, or when another teacher was absent or had completed their syllabus. This improved in the second year, when only one of the school principals had not allotted a timetable slot to the life skills programme. While those SHCs who did not have a reserved period still managed to find ways to deliver most of the life skills syllabus, some of the SHCs and programme staff faced continued challenges in finding suitable space for counselling.

"*“Infrastructure is the problem of the school because they don’t have proper place for their own classes so we cannot demand for a class or place.”*"

Female Clinical Supervisor 1, 2010–11

A supportive and enthusiastic school management team was identified by programme coordinators as the most important facilitator, as this could reduce the burden of organisation and motivation carried by the SHC, and improve the participation of all school members in the programme activities.

"*“Actually some schools gave us a class and some schools did not. Some schools talked about after class. Some few schools gave us the library period or [physical education] period. You know, I think this was a lot determined by the principals. So I can’t say that across the board everybody gave us a class…it was a lot dependent on the principals and how important they thought the programme was.”*"

Female intervention coordinator, 2010–11

### **Acceptability**

The acceptability of the programme activities was assessed quantitatively in terms of the uptake of services offered. Increasing acceptability of the programme over the two years was indicated by the growing number of submissions to the anonymous letter box for student complaints and questions, from 220 in the first year to 770 in the second year. In 2010–11, the majority of the questions related to psycho-social issues (49.8%), followed by students’ complaints and concerns relating to school infrastructure, management and administration (36.9%).

A second indicator of acceptability was the increasing number and proportion of students who sought individual counselling on their own and returned for follow up counselling sessions in the second year. While the total number of students who accessed individual counselling was stable across the two years (just over 6% of the student population), growing acceptability was reflected in the increased number of counselling sessions (from 251 in 2009–10 to 323 in 2010–11), and the increased proportion of self-referred cases, from 8.2% in the first year to 24.2% in the second year.

A review of stakeholder perceptions and experiences of the programme provided qualitative evidence about the acceptability of the intervention activities and of the SHC as an agent of delivery. There was a high level of expressed support for most of the programme activities from principals, teachers and students. For example, the school management, teachers and the students all saw the nutritional screening and vision assessment camps as an important means of identifying and addressing common health problems amongst populations with limited access to paid health care. In the words of one student:

"*“Some students cannot afford to go to doctors for these types of health check-ups. These people are doing this in our school, and that too free of cost. That is what I like the most.”*"

*9*^*th*^*Grade Female Student, School H, 2009–10*

The anonymous letter box for student questions and concerns was also considered an important outlet for students by all stakeholder groups. According to students, the factors that increased acceptability were anonymity, confidentiality and the SHCs’ efforts to address student concerns. For example:

"*“I like the Speak-Out Box because students can get their problems solved without others coming to know.”*"

*9*^*th*^*Grade Male Student, School H, 2009–10*

"*“What we cannot share with our friends we can write and share with [the school health counsellor], who will keep it a secret. I liked that.”* (11^th^ grade female student)"

*11*^*th*^*Grade Female Student, School I, 2009–10*

While all of the school management and most of the staff interviewed were supportive of the Speak-Out Box, one teacher mentioned his reservation that students might make false reports about teachers they do not like, while another teacher felt that the lay school health counsellor was not equipped to deal with the problems raised through the Speak-Out Box. This appeared to be related to students in those schools having complained about teachers’ disciplinary practices, and the feedback being escalated by the school health counsellor to the principal.

All the stakeholder groups mentioned positive attitudes towards the life skills programme, noting that it was useful and relevant. However, in 4 out of 10 schools, the reproductive and sexual health sessions were not taught because the school management felt that the topic was not appropriate. Many students spoke about enjoying the interactive and activity-based nature of the teaching. The school principals and SHCs summarised a range of factors that contributed to the success of the life skills programme: these included consultation with the principal on the syllabus; age appropriate modules; interactive sessions, and relevant materials. In the first year, several of the teachers had recommended greater use of visual aids, which was incorporated in the second year.

In the first year of the programme, the SHCs and principals spoke about challenges to the uptake of the individual counselling, such as stigmatization of the service by students and teachers’ disappointment with the results, may have been linked to unrealistic expectations of the process and outcomes. In the second year, teachers were sensitized by the SHCs about the purpose of counselling and the type of results to be expected, and the interviews with SHCs and teachers indicated more appropriate referrals for counselling and teacher satisfaction with the outcomes. At the same time, the SHCs noted that the students were more forthcoming about attending the counselling sessions and opening up about their problems. Principals, SHCs and the programme coordinators attributed the increased student uptake and participation to greater awareness of the benefits of counselling, and students’ growing confidence that information would be kept confidential. For example:*Female Clinical Supervisor 3, 2010–11Male Principal, School E, 2010–119*^*th*^*Grade Female Student, School A, 2010–11*

"*“In the beginning, yes…counselling was seen as stigmatising, when teachers kept sending cases. But slowly when children started understanding that this counsellor is part of the school and they can go to her with any problem, then there was less stigma, students started coming on their own.”*"

"*“Initially counselling was a very new concept to them and they (students) may have had some misunderstanding about the things, but now the thing is they have realized the importance of it.”*"

"*“Earlier we used to feel that the [counsellor] may tell others whatever we tell her so we were scared to speak. But because of counselling, our doubts were cleared and we got help in dealing with our problems. [Now] we know that [the counsellor] doesn’t share this information with anyone else.”*"

The students’ and principals’ perceptions of the SHC as an agent of delivery were favourable. Many students talked of their ability to open up and engage with the SHC, because she was more approachable than their other teachers. For example:*6*^*th*^*Grade Male Student, School B, 2009–10*

"*“Initially, we were not telling our problems to our teachers since they would get angry, but now we are telling our [SHC].”*"

In the first year of the programme, three teachers who were interviewed mentioned that they felt that the SHC was not mature enough to deliver all of the programme activities. Several teachers also complained about a lack of information sharing about the programme; this was addressed in the second year by monthly reports to teachers by the SHCs. The SHCs reported that this had resulted in greater teacher support, which appeared to be corroborated by reduced negative feedback from teachers.

### **Impact**

Preliminary impact of the programme was indicated quantitatively by the number of students who were identified with health problems in the nutritional screening and visual assessment camps and the number who received any follow up or treatment, and qualitatively through evidence from semi-structured interviews about the effects of the various programme activities on student health and wellbeing. The health camps were a major activity conducted by the SHCs, with all eligible students present on the day/s of the camp being screened for nutritional status and full vision. In 2009–10, and 2010–11, nutritional and visual assessment camps were conducted in all schools, with the majority of eligible students being screened. In the first year, there was some criticism from principals that the health camps had not resulted in adequate follow up, even where problems were identified. In the second year, however, there was a consensus across stakeholders that the camps were more effective, as many of the students who were identified with visual or nutritional problems received follow up as a result of the SHCs’ efforts.

In 2010–11, 1,987 students underwent visual screening. Out of these students, 443 were found to have probable refractory error and 100 were detected with probable colour blindness. These students were referred for confirmation of diagnosis and treatment; 223 of the 512 students who were referred (43.6%) visited a health care provider. One of the school teachers spoke about the benefits of the visual assessment camp:*Female Teacher, School C, 2010–11*

"*“After the check up, [the SHC] did the follow up and children were given spectacles, which I had not expected. It was a really good initiative to give them spectacles because most of the students in our school are poor and they may not have been able to afford to buy the spectacles.”*"

Out of 1,917 students who received a nutritional assessment in 2010–11, 653 (34.1%) were found to be underweight and 134 (7%) were overweight or obese. These students received dietary guidelines to be taken home and shared with their parents. A parent commented on the positive effect of the nutritional assessment on her child:*Female Parent, School C, 2010–11*

"*“[My son] was under weight, [the SHC] gave us a chart in which it is written what to eat and in what quantity to eat, and my son is following this. First I used to have to force him to eat, but now after this counsellor gave him a book about what to eat…he forces me to cook all the things that are there in this book for him.”*"

The Speak Out Box was a school-wide intervention that resulted in action on a range of issues. The submissions could be anonymous and were reviewed on a weekly basis by the SHC who took the issues up with the school management where appropriate. In several schools, problems in the health environment were raised. The SHCs and principals related several instances in which the school management had acted in response to student complaints: for example, fans and drinking water filters were installed, furniture was fixed, and regular cleaning of common facilities was enforced in some schools. Other relevant Speak Out Box issues that were addressed by the school included two cases of teachers’ extreme disciplining practices and many cases of student bullying. Anti-bullying workshops/sessions were held in all schools for the management and the students. Questions about reproductive and sexual health and life skills were frequently raised through the Speak Out Box, and were addressed by the SHC in classroom based life skills sessions.

Teachers, students and parents mentioned a range of positive effects of the life skills educational sessions. One teacher discussed substance use amongst students:*Female Teacher, School A, 2010–11*

"*“Earlier some students used to [chew tobacco], which is no longer happening, and also gambling was there before, but this year we have not found any students with such practices…They are aware of the consequences.”*"

Behavioural improvements were also noted by some parents. For example:*Female Parent, School A, 2010–11*

"*“There has been a lot of effect on the children, earlier they would not give up their places on the bus for people to sit. Now they get up and give their seats, especially for elders. Earlier my daughter would not listen and used to back answer me, but now there is lot of improvement in her, she behaves well. Ever since [the SHC] spoke to her, she has not back answered me.”*"

Students from across the schools and grades mentioned that they were using the knowledge and skills learned in the classroom programme in their daily lives. This included adopting healthy eating habits (for example, reduction in consumption of aerated beverages), being more respectful to elders and more civil to peers (i.e. not bullying), avoiding abusive language, changing attitudes towards education, becoming more aware of personal safety and safety in the community, managing anger better, improving personal hygiene, and understanding the physiological changes associated with adolescence. Additionally, many students said that they had benefited from the study skills sessions, and were following the tips for concentration, memorisation and making a study timetable. In some cases, this had led to tangible improvements in academic outcomes. For example:*6*^*th*^*Grade Male Student, School F, 2009–10*

"*“[the SHC] used to tell us what should be done in the exams and all so I followed that and I passed in many papers. First I had failed in two papers which I used to find difficult, but now I passed.”*"

Finally, all groups of stakeholders recounted examples of the positive impact of individual counselling on students. In the first year, the SHCs complained about many inappropriate referrals by teachers who were not able to discipline their classes, as counselling could not be a substitute for better classroom disciplining practices. This was less of a concern in the second year, with teachers’ and students’ increased awareness about the goals of counselling and a higher proportion of self-referred cases. SHCs, students, and teachers spoke of instances where SHCs, sometimes supported by the clinical psychologists, were able to help students to cope with and/or solve their problems. These included cases of harassment and abuse, domestic violence, educational difficulties, defiant behaviour, bullying, and relationship issues. For example:*7*^*th*^*Grade Female Student, School A, 2010–201110*^*th*^*Grade Male Student, School H, 2009–10Female Principal, School C, 2010–11Female Parent, School C, 2010–11Male Parent, School E, 2010–11*

"*“I opened up my secret to (the SHC), which I had never done before, not even at home. She helped me to solve it and now I am fine. Now everyone is good to me they all smile at me or speak to me. I liked it.”*"

"*“I was a repeater in the 9*^*th*^*standard. [The SHC] taught me study skills and gave me a time table in writing so I followed that time table and I passed and now I am in 10*^*th*^*standard.”*"

"*“[One] child was involved with a boy. Only child of the parents and she used to be very disturbed in the class also and the counsellor did help her. And this child has really come out of the problem and she has assured that she will take care of herself and her studies at this moment and not be disturbed with all this.”*"

"*“My younger son was very short tempered, they spoke to him. That anger he had learned from me, because I used to get angry very quickly and he is just like me. Now my son is under control, they explained to him how to control his anger.”*"

"*“There used to be complaints about my son from all teachers in all classes. Now after counselling there are no complaints about him from teachers.”*"

While there were many examples of success in individual counselling, clinical supervisors and SHCs identified some constraining factors which could limit its effectiveness, including the absence of a specific time for counselling in some schools, teachers’ stigmatising attitudes, and the limited capacities of SHCs to deal with serious cases. At the end of the second year, one of the three clinical supervisors mentioned some reservations about the SHC capacities to undertake individual counselling for difficult cases. The other two clinical supervisors reported a substantial improvement in the counselling skills of the SHCs and their ability to handle cases independently, and felt that the supervision and joint case consultation had been an effective training mechanism for the majority of cases seen. The intervention coordinator noted that all the counsellors were able to deal with cases relating to academic concerns (study skills, exam anxiety, detection of learning disorders, and career counselling); with psychosocial issues such as bullying and disability; and, with physiological concerns such as nutrition. A few SHCs continued to seek support for dealing with cases related to RSH, relationships, and suicidal ideation. Clinical psychologists mentioned that it would be important to provide on-going supervision/training for SHCs for continued skills development, as well as to ensure clearly defined referral pathways for students who need support from certified professionals.

## **Discussion**

We report on the development of a lay counsellor delivered multi-component Health Promoting Schools intervention in 10 schools in Goa. We observed that a high coverage of planned activities was achieved over the first two years. Despite reduced supervision of the school health counsellors (SHCs) from the first to the second year of the intervention, the coverage (proportion of target delivered) of programme activities increased. The activity in which the SHCs appear to require the most supervision was individual counselling; but even for this component, the numbers of adolescents counselled remained stable, self-referrals grew and follow-up sessions increased. An increasing proportion of sessions were carried out by the SHC without supervision in the second year of the programme.

Several changes were made in the second year of the intervention in response to feedback from the mid-term evaluation, including modifications in the classroom module content and mode of delivery; provision of regular information to teachers about the activities; ensuring a classroom and teaching period for the life skills session; and, securing a quiet and comfortable space for individual counselling. Overall acceptability of the activities in both years was indicated by the high uptake of services. Additionally, there was widespread agreement amongst stakeholders of the need for the programme in order to improve the health, wellbeing, and academic outcomes of students. Only one out of the ten principals was not fully cooperative, and a minority of teachers expressed persisting misconceptions about the role of the counsellor and a lack of sensitivity towards adolescents who were accessing the individual counselling service. The impact of the intervention was indicated quantitatively by the substantial number of students who were identified with and treated for nutritional or visual problems. There was also strong qualitative evidence of changes in the school environment as a result of the Speak-Out Box; in knowledge and behaviour of students as a result of life skills sessions; and, in academic, social, and mental health outcomes of students who accessed individual counselling.

We identified two other robust evaluations of school health programmes in India, both of which have yielded promising results. One teacher and peer-delivered intervention to reduce tobacco use found that students who received a school and family based intervention were significantly less likely than students in the control group to have used or have intentions to use tobacco [30]. The second, a Health Promoting Schools intervention delivered by health care providers in rural India, reported significant improvements in the nutritional status and personal hygiene of students, with associated reductions in hygiene related health problems [16]. School health promotion programmes in India have generally been developed for delivery by teachers, health care providers, or peers, partly because this is perceived as a low cost option. However, these programmes can compete with teachers’ or health care providers’ other time commitments. Peer education programmes contend with a constantly shifting population of student peer educators; thus, the sustainability and, consequently, effectiveness, of these delivery models may be limited [31]. The scalability of programmes delivered by dedicated teachers or health professionals in India is likely to be constrained by the limited number of such professionals in the country and their salary costs.

A lay health counsellor presents a potentially sustainable and scalable alternative approach for school health promotion. There are, however, no previous published studies from India or other Low or Middle Income Countries of process evaluations of multi-component school health interventions delivered by lay health workers. Formative and piloting research that incorporates mixed methods is widely recognised as critical for understanding the pathways to effectiveness of school health interventions [32,33]. Much of the literature in this field is drawn from high income settings, including the United Kingdom, the United States, Europe, Australia and New Zealand [34-37] and our study is one of the few from a developing country describing the process of development of a school based health intervention and of task-shifting the delivery of health promotion activities to a lay health counsellor.

### **Limitations of the study**

There are some limitations to the study design. First, the intervention was conducted in government aided schools in only one state of India, and the school environment may not be generalizable across all school types and states; however, the findings may be extrapolated, with appropriate caution, to government schools in other urban and peri-urban settings, as they share similar socio-economic characteristics as Goa. Second, despite a reduction in the intensity of supervision from the first to second year of the programme, the NGO played an important monitoring role; this level of supervision may not be feasible or affordable in a scaled up programme, and could influence the quality of delivery. Third, as this was a case study and did not include a control group or statistically powered study with quantitative outcome measures, our ability to draw inferences about the effectiveness of the intervention in changing students’ knowledge or academic/health outcomes is limited. In fact, this study was designed in the context of the formative and piloting stages of the development of a complex intervention [38]. The primary aim was to assess whether the model of health promotion and the method of delivery was acceptable, feasible and showed promise of effectiveness. Thus, this study reports the first stage of evidence generation, with a focus on development, refinement and feasibility testing, which may lead to further quantitative testing of the effectiveness of the intervention.

## **Conclusion**

The feasibility, acceptability and preliminary evidence indicative of impact of the lay school health counsellor delivered HPS programme suggest that this could be a promising model for definitive evaluation, and for subsequent scaling up of school health promotion, particularly where qualified teachers and health professionals are in short supply. The encouraging findings of this study warrant further research to generate evidence of the effectiveness and cost-effectiveness of different human resource delivery models for health promotion interventions in schools, comparing the SHC led intervention described in this paper with traditional models, such as teacher, peer, and psychologist led delivery. Such information would be critically important to influence wider programme and policy decisions about school health promotion in India, and in other resource limited settings.

## **Competing interests**

The authors declare that they have no competing interests.

## **Authors’ contributions**

DR contributed in the design of the evaluation framework, database development, data analysis, and drafting the manuscript. ST contributed in the design of the evaluation framework, database development, data collection, data quality monitoring, data analysis, and review of the manuscript. AC contributed in the design of the intervention and the evaluation framework, and review of the manuscript. BB contributed in the design of the evaluation framework, database development, data collection, data analysis, and review of the manuscript. GA contributed in the design of the evaluation framework, data analysis, and review of the manuscript. SP contributed in database development, data collection, data quality monitoring, data entry and data analysis, and review of the manuscript. VP contributed in the design of the intervention and the evaluation framework, database development, data analysis, and drafting the final manuscript. All authors read and approved the Final manuscript.

## Pre-publication history

The pre-publication history for this paper can be accessed here:

http://www.biomedcentral.com/1472-6963/12/127/prepub
